# Supplementary material for: Molecular Modeling Studies to Probe the Binding Hypothesis of Novel Lead Compounds against Multidrug Resistance Protein ABCB1
Source: Biomolecules. 2024 Jan 16;14(1):114. doi: 10.3390/biom14010114 (PMC10813284; doi:10.3390/biom14010114)
Supplement: Supplementary file 1 [file biomolecules-14-00114-s001.zip › biomolecules-2768650-supplementary.pdf]

Table S1. ABCB1-inhibitors retrieved from the literature and recently reported leads [1] [2] [3] [4] [5] [6] along with their IC_{50}, pIC_{50} values, clogP, LipE, molecular weight and number of heavy atoms. *NA (Not Active), *ND (Not Determined). Cs A, cyclosporine A ; Tar, tariquidar; Ela, Elacridar; Zos, Zosuquidar.

**Compounds**	**IC_{50} [µM]**			**pIC_{50}**			**clogP**	**LipE**			**Weight**	**heavy atom**	**References**
	**ABCB1**	**ABCG2**	**ABCC1**	**ABCB1**	**ABCG2**	**ABCC1**		**ABCB1**	**ABCG2**	**ABCC1**			
1A	0.78	NA	NA	6.11	ND	ND	4.37	1.74	ND	ND	533.64	39	[1]
2A	0.30	NA	NA	6.52	ND	ND	5.19	1.33	ND	ND	567.71	42	[1]
3A	0.70	NA	NA	6.15	ND	ND	5.56	0.59	ND	ND	505.64	38	[1]
4A	0.70	NA	NA	6.15	ND	ND	4.13	2.02	ND	ND	531.63	39	[1]
5A	1.04	NA	NA	5.98	ND	ND	4.02	1.96	ND	ND	507.61	37	[1]
6A	0.66	NA	NA	6.18	ND	ND	6.52	-0.34	ND	ND	517.65	39	[1]
7B	1.40	NA	NA	5.85	ND	ND	5.03	0.82	ND	ND	534.63	39	[1]
8B	0.33	NA	NA	6.48	ND	ND	5.83	0.65	ND	ND	568.69	42	[1]
9B	10.00	NA	NA	5.00	ND	ND	6.20	-1.20	ND	ND	506.62	38	[1]
10B	2.00	10	NA	5.70	5	ND	4.57	1.13	0.43	ND	532.61	39	[1]
11B	0.93	17	NA	6.03	4.7	ND	4.74	1.29	-0.04	ND	508.59	37	[1]
12B	1.23	5.9	NA	5.91	5.2	ND	7.49	-1.58	-2.29	ND	518.63	39	[1]
13C	0.68	NA	NA	6.17	ND	ND	4.94	1.23	ND	ND	519.66	38	[1]
14C	0.57	NA	NA	6.24	ND	ND	6.32	-0.08	ND	ND	553.72	41	[1]

15C	0.73	NA	NA	6.14	ND	ND	4.01	2.13	ND	ND	493.62	36	[1]
16C	1.01	NA	NA	6.00	ND	ND	6.96	-0.97	ND	ND	503.67	38	[1]
17D	20.00	ND	>100	4.70	ND	>4	3.50	1.20	ND	>0.5	366.21	22	[4]
18D	7.20	ND	>100	5.14	ND	>4	3.15	1.99	ND	>0.85	378.43	28	[4]
19D	1.40	ND	>100	5.85	ND	>4	4.06	1.79	ND	>-0.06	381.40	28	[4]
20D	1.90	ND	>100	5.72	ND	>4	3.19	2.53	ND	>0.81	420.47	31	[4]
21D	0.57	ND	>100	6.24	ND	>4	4.10	2.14	ND	>-0.1	408.41	30	[4]
22D	17.30	ND	>100	4.76	ND	>4	2.01	2.75	ND	>1.99	416.45	29	[4]
23D	6.80	ND	6.1	5.17	ND	5.21	2.63	2.54	ND	2.58	409.40	30	[4]
24D	2.60	ND	2.5	5.59	ND	5.60	1.69	3.90	ND	3.91	420.37	30	[4]
25D	0.20	ND	>100	6.70	ND	>4	4.07	2.63	ND	>-0.07	452.42	33	[4]
26D	0.60	ND	>100	6.22	ND	>4	3.55	2.67	ND	>0.45	450.45	33	[4]
27D	24.30	ND	11.2	4.61	ND	4.95	3.40	1.21	ND	1.55	498.49	36	[4]
28D	0.40	ND	>100	6.40	ND	>4	4.13	2.27	ND	>-0.13	408.41	30	[4]
29D	1.30	ND	>100	5.89	ND	>4	4.06	1.83	ND	>-0.06	378.38	28	[4]
30D	6.60	ND	>100	5.18	ND	>4	4.61	0.57	ND	>-0.61	378.38	28	[4]
31D	1.50	ND	>100	5.92	ND	>4	4.92	1.00	ND	>-0.92	394.43	29	[4]
32D	2.00	ND	>100	5.70	ND	>4	3.12	2.58	ND	>0.88	408.41	30	[4]
33D	14.10	ND	30.0	4.85	ND	4.52	3.67	1.18	ND	0.85	353.35	26	[4]
34D	3.50	ND	>100	5.46	ND	>4	2.38	3.08	ND	>1.62	365.36	27	[4]

35E	0.05	ND	15.1	7.30	ND	4.82	5.05	2.25	ND	-0.23	515.63	38	[3]
36I	1.51	37.90	ND	5.82	4.42	ND	3.64	2.18	0.72	ND	342.46	25	[5]
37I	1.70	33.50	ND	5.77	4.47	ND	4.23	1.54	0.24	ND	356.49	26	[5]
38I	0.80	30.70	ND	6.10	4.51	ND	4.38	1.72	0.13	ND	368.50	27	[5]
39I	1.56	31.90	ND	5.81	4.50	ND	4.61	1.20	-0.11	ND	326.46	24	[5]
40I	0.65	36.40	ND	6.19	4.44	ND	4.85	1.34	-0.41	ND	383.53	28	[5]
41I	0.65	15.20	ND	6.19	4.82	ND	5.06	1.13	-0.24	ND	399.57	29	[5]
42I	3.01	37.30	ND	5.52	4.43	ND	4.23	1.29	0.20	ND	385.55	28	[5]
43I	0.27	4.62	ND	6.57	5.34	ND	5.27	1.30	0.07	ND	462.57	34	[5]
44I	0.09	13.90	ND	7.05	4.86	ND	5.49	1.56	-0.63	ND	478.61	35	[5]
45I	0.79	14.10	ND	6.10	4.85	ND	4.79	1.31	0.08	ND	434.51	32	[5]
46I	113.00	440.00	ND	3.95	3.36	ND	1.39	2.56	1.97	ND	293.36	21	[5]
47I	5.83	34.00	ND	5.23	4.47	ND	2.96	2.27	1.51	ND	370.47	27	[5]
48I	3.60	47.10	ND	5.44	4.33	ND	3.82	1.62	0.51	ND	354.47	26	[5]
49I	1.79	600.00	ND	5.75	3.22	ND	2.01	3.74	1.17	ND	370.47	27	[5]
50I	0.68	58.80	ND	6.17	4.23	ND	2.58	3.59	1.65	ND	384.50	28	[5]
51I	0.16	98.30	ND	6.80	4.01	ND	3.65	3.15	0.36	ND	432.54	32	[5]
52I	0.39	3.30	ND	6.41	5.48	ND	6.11	0.30	-0.63	ND	479.62	36	[5]
53I	5.50	4.21	ND	5.26	5.38	ND	4.45	0.81	0.93	ND	375.47	28	[5]
54I	9.61	1.50	ND	5.02	5.82	ND	4.97	0.05	0.85	ND	433.50	32	[5]

A	0.001	ND	ND	8.82	ND	ND	3.13	5.69	ND	ND	374.49	28	[6]
D	0.019	ND	ND	7.72	ND	ND	2.35	5.37	ND	ND	388.38	27	[6]
E	0.01	ND	ND	8	ND	ND	1.99	6.01	ND	ND	352.38	26	[6]
F	0.003	ND	ND	8.42	ND	ND	2.53	5.89	ND	ND	450.41	30	[6]
CsA	1.2	26.1	6.6	5.92	4.58	5.18	14.36	-7.37	ND	ND	1202	85	[7] [8]
Tar	0.03	0.9	NA	7.48	6.05	ND	5.55	1.93	ND	ND	646.47	48	[9]
Ela	0.07	10	NA	7.14	5.0	ND	4.21	2.93	ND	ND	563.60	42	[9] [1]
Zos	0.05	ND	ND	7.23	ND	ND	4.96	2.27	ND	ND	527.00	39	[9]

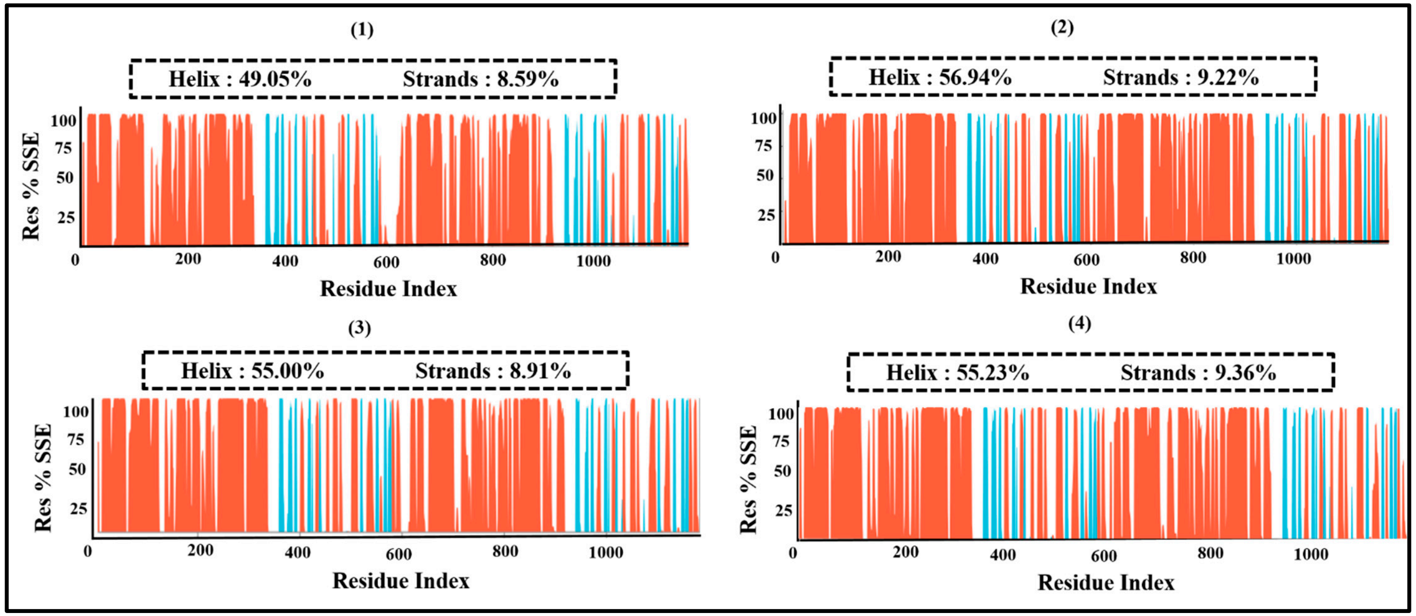

Figure S1. Protein Secondary Structure Elements (SSE) like helices and strands are scrutinized throughout the simulation of 100 ns. The plot above reports percentage SSE distribution by residue index throughout the protein structure. **(1)** 'ABCB1-A' complex; **(2)** 'ABCB1-D' complex; **(3)** 'ABCB1-E' complex; **(4)** 'ABCB1-F' complex along with the percentage of helices and strands, displayed with the plot of each complex.

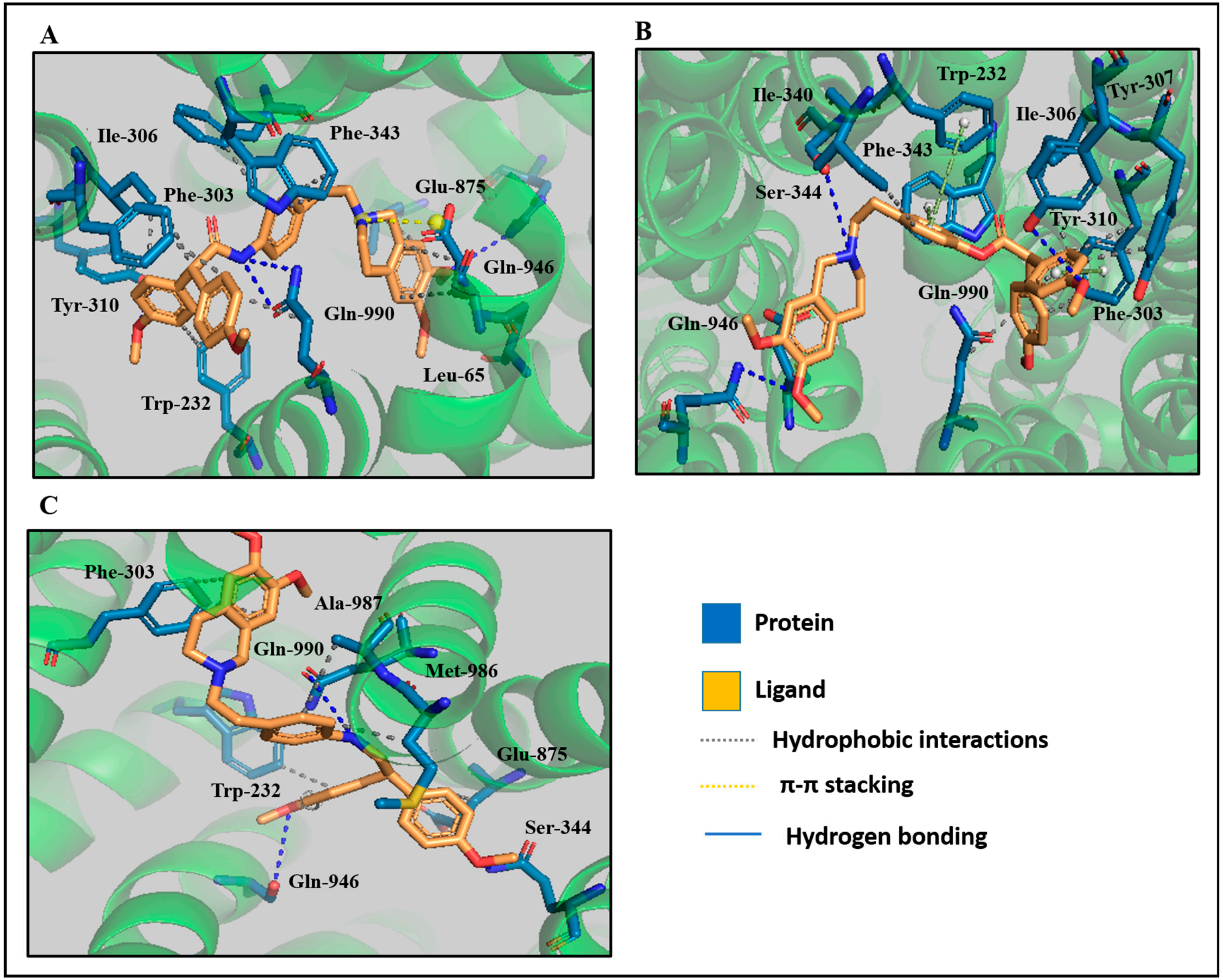
A
Ile-306
Phe-343
Phe-303
Glu-875
Gln-946
Tyr-310
Gln-990
Leu-65
Trp-232
B
Trp-232
Ile-340
Tyr-307
Ile-306
Phe-343
Ser-344
Tyr-310
Gln-990
Gln-946
Phe-303
C
Phe-303
Ala-987
Gln-990
Met-986
Glu-875
Trp-232
Ser-344
Gln-946
Protein
Ligand
Hydrophobic interactions
π-π stacking
Hydrogen bonding

Figure S2. Compounds and the interacting residues are shown in stick form. **(A)** Compound '2A' showed hydrophobic interactions with Leu-65, Trp-232, Phe-303, Ile-306, Glu-875, and Gln-990 while hydrogen bonding with Tyr-310, Gln-946, and Gln-990 **(B)** Compound '8B' showed hydrophobic interactions with Phe-303, Ile-306, Tyr-307, Ile-340, and Gln-990, hydrogen bonding with Tyr-310, Ser-344 and Gln-946 and π stacking with Trp-232, Phe-303 and Phe-343. **(C)** Compound '14C' showed hydrophobic interactions with Leu-65, Trp-232, Phe-303, Glu-875, Met-986, Ala-987 while hydrogen bonding with Ser-344, Gln-946 and Gln-990.

Table S2. The derivatives of 6,7-dimethoxy-2-phenethyl-1,2,3,4-tetrahydroisoquinoline with the substituent groups at R[1] and activity values against ABCB1 and ABCC1 [1].

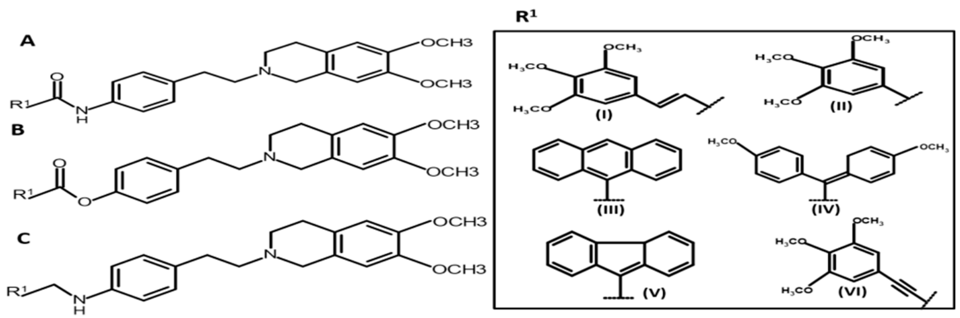

**Compounds**	**Scaffold**	**R^1**	**IC$_{50}$(ABCB1)** [µM]	**IC$_{50}$(ABCC1** [µM])	**IC$_{50}$(ABCG2** [µM])
1A	A	I	0.78	Not Active	Not Active
2A	A	IV	0.30	Not Active	Not Active
3A	A	V	0.70	Not Active	Not Active
4A	A	VI	0.70	Not Active	Not Active
5A	A	II	1.04	Not Active	Not Active
6A	A	III	0.66	Not Active	Not Active
7B	B	I	1.40	Not Active	Not Active
8B	B	IV	0.33	Not Active	Not Active
9B	B	V	10	Not Active	Not Active

10B	B	VI	2.0	Not Active	10
11B	B	II	0.93	Not Active	17
12B	B	III	1.23	Not Active	5.9
13C	C	I	0.68	Not Active	Not Active
14C	C	IV	0.57	Not Active	Not Active
15C	C	II	0.73	Not Active	Not Active
16C	C	III	1.01	Not Active	Not Active

Table S3. The galloyl benzamide derivatives with the substituent groups at with the different substituent at R[1], R[2], R[3], R[4], R[5], R[6], X and Y position along with the activity values against ABCB1 and ABCC1 [4] [3].

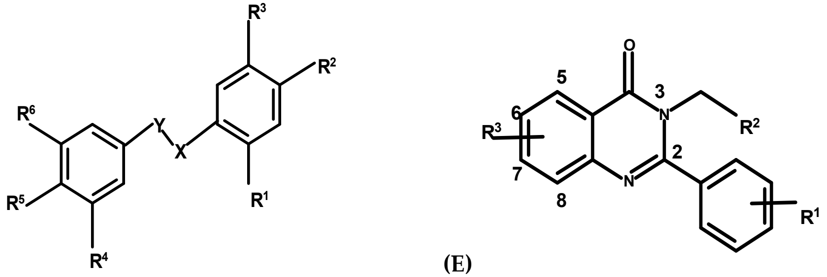

(D) (E)

Compounds	X	Y	R¹	R²	R³	R⁴	R⁵	R⁶	IC₅₀(ABCB1) [μM]	IC₅₀(ABCC1 [μM])
17D	NH	CO	H	Br	H	OCH_3	OCH_3	OCH_3	20	>100
18D	NH	CO	NH2	Ph	H	OCH_3	OCH_3	OCH_3	7.2	>100
19D	NH	CO	F	Ph	H	OCH_3	OCH_3	OCH_3	1.4	>100
20D	NH	CO	$NHCOCH_3$	Ph	H	OCH_3	OCH_3	OCH_3	1.9	>100
21D	NH	CO	NO_2	H	Ph	OCH_3	OCH_3	OCH_3	0.57	>100
22D	NH	CO	NO_2	2-thienyl	H	OCH_3	OCH_3	OCH_3	17.3	>100

23D	NH	CO	NO_2	3-pyridyl	H	OCH_3	OCH_3	OCH_3	6.8	6.1
24D	NH	CO	NO_2	3,5-difluorophenyl	H	OCH_3	OCH_3	OCH_3	2.6	2.5
25D	NH	CO	NO_2	Benzo[1,3]dioxol-5-yl	--	OCH_3	OCH_3	OCH_3	0.2	>100
26D	NH	CO	NO_2	3-acetophenyl	H	OCH_3	OCH_3	OCH_3	0.6	>100
27D	NH	CO	NO_2	3,4,5-$(OCH_3)_3$Ph	H	OCH_3	OCH_3	OCH_3	24.3	11.2
28D	NH	CO	H	3-nitrophenyl	H	OCH_3	OCH_3	OCH_3	0.4	>100
29D	NH	CO	NO_2	Ph	H	H	OCH_3	OCH_3	1.3	>100
30D	NH	CO	NO_2	Ph	H	OCH_3	H	OCH_3	6.6	>100
31D	NH	CH_2	NO_2	Ph	H	OCH_3	OCH_3	OCH_3	1.5	>100
32D	CO	NH	NO_2	Ph	H	OCH_3	OCH_3	OCH_3	2.0	>100
33D	NH	CO	F	Ph	H	OH	OH	OCH_3	14.1	30
34D	NH	CO	NO_2	Ph	H	H	OCH_3	OH	3.5	>100
35E	-	-	4-N(CH3)2	2-ethyl-6,7-dimethoxy-1,2,3,4-tetrahydroisoquinoline	8- OCH3	-	-	-	0.05	15.1

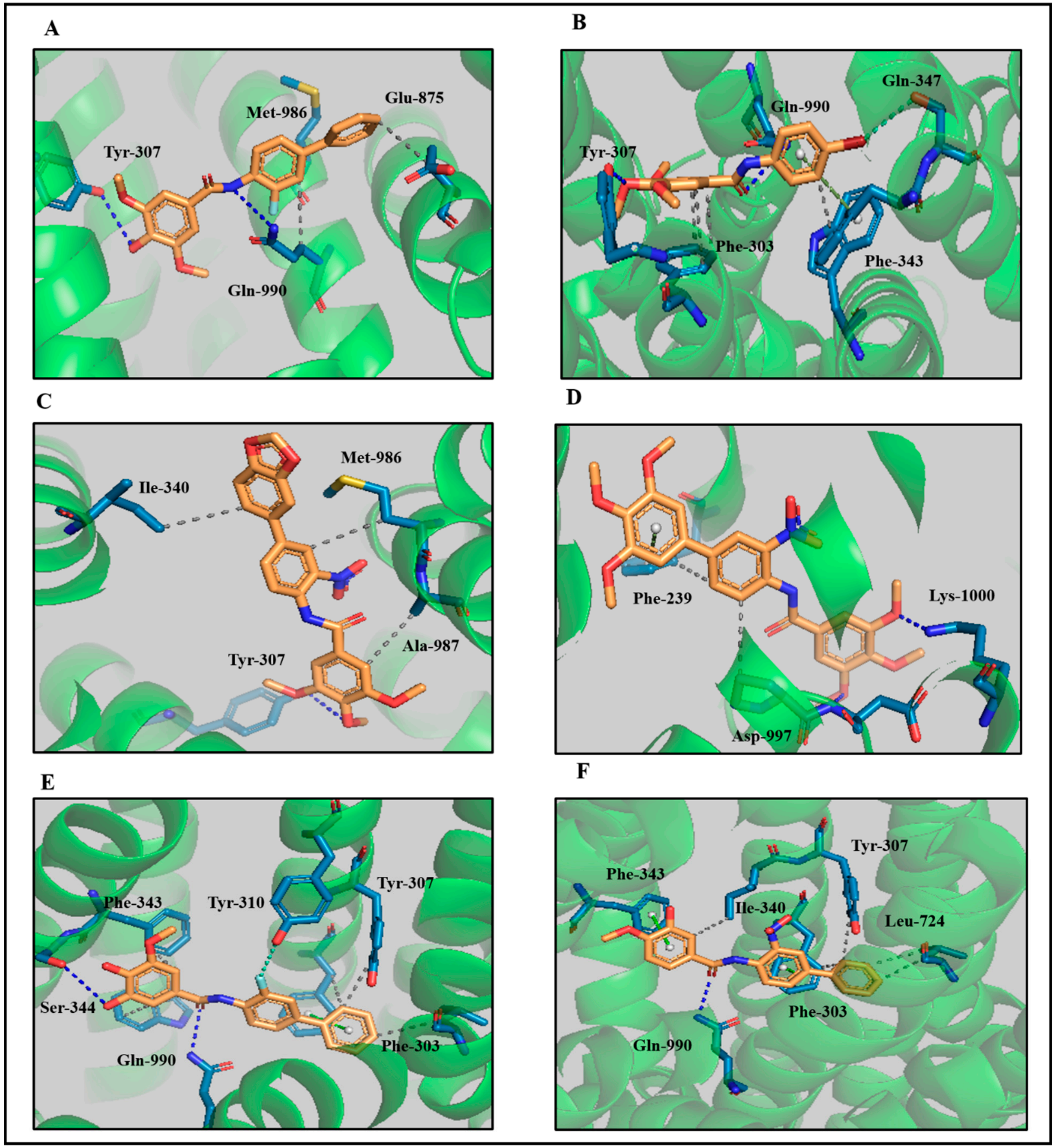
A
Tyr-307
Met-986
Glu-875
Gln-990
B
Gln-347
Gln-990
Tyr-307
Phe-303
Phe-343
C
Ile-340
Met-986
Ala-987
Tyr-307
D
Phe-239
Lys-1000
Asp-997
E
Phe-343
Tyr-310
Tyr-307
Ser-344
Gln-990
Phe-303
F
Tyr-307
Phe-343
Ile-340
Leu-724
Phe-303
Gln-990

x

Figure S3. Compounds and interacting residues are shown in stick form. **(A)** Compound '19D' showed hydrophobic interactions with Glu-875, Gln-990, and Met-986 while hydrogen bonding with Tyr-307 and Gln-990 **(B)** Compound '17D' showed hydrophobic interactions with Phe-303, Tyr-307, and Gln-347, hydrogen bonding with Gln-990 and π stacking with Phe-343. **(C)** Compound '25D' showed hydrophobic interactions with Ile-340, Met-986 and Ala-987 while hydrogen bonding with Tyr-307. **(D)** Compound '27D' showed hydrophobic interactions with Phe-239 and Asp-997 while hydrogen bonding with Lys-1000. **(E)** Compound '33D' showed hydrophobic interactions with Phe-303, Tyr-307, Phe-343 and Ser-344 while hydrogen bonding with Gln-990 and π stacking with Tyr-310. **(F)** Compound '34D' showed hydrophobic interactions with Phe-303, Ile-340, Phe-343, and Leu-724 while hydrogen bonding with Gln-990.

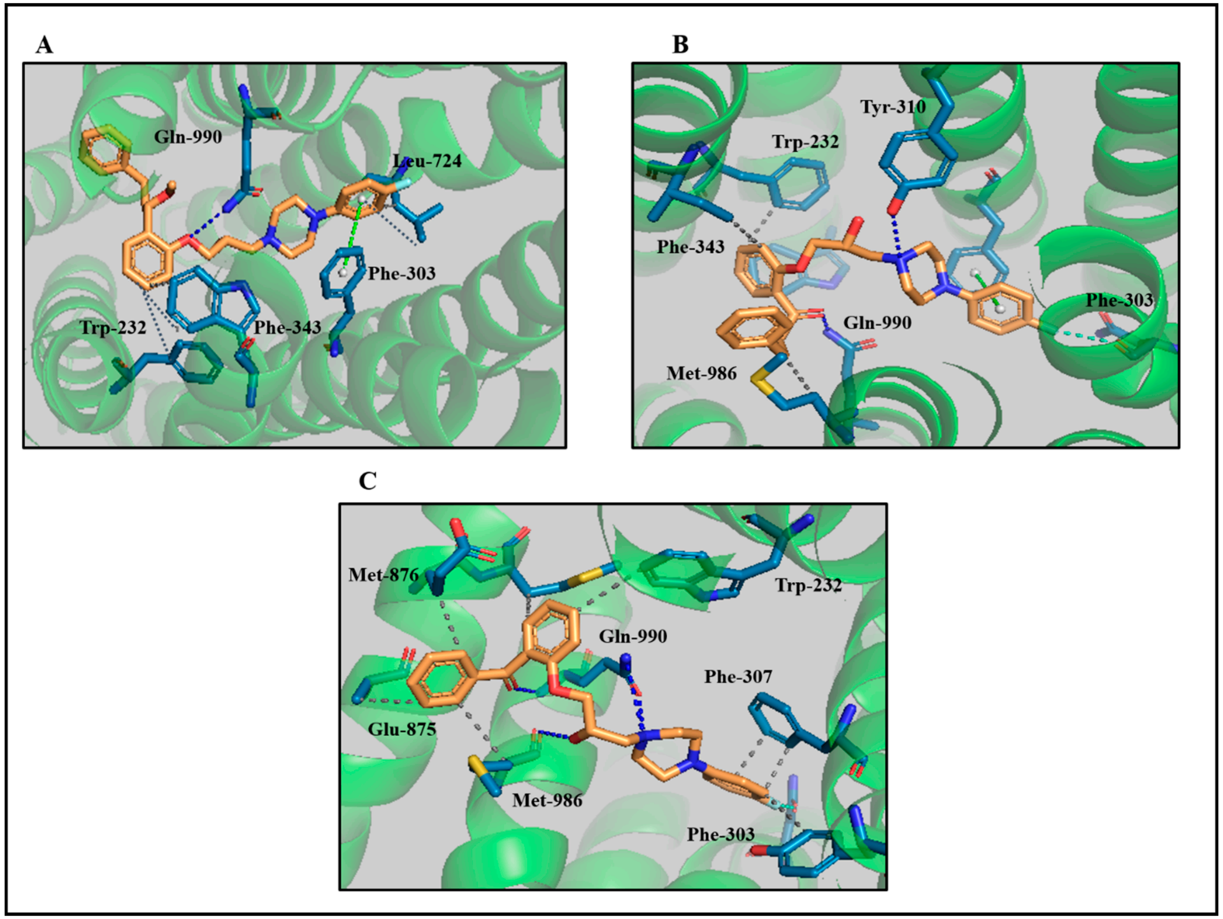

Figure S4. Compounds and interacting residues are shown in stick form. **(A)** Compound ‘44I’ showed hydrophobic interactions with Trp-232, Phe-343, and Leu-724 while π stacking with Phe-303 and hydrogen bonding with Gln-990. **(B)** Compound ‘43I’ showed hydrophobic interactions with Trp-232, Tyr-310, Phe-343, Met-986, and Met-876, hydrogen bonding with Gln-990 and Phe-303. **(C)** Compound ‘45I’ showed hydrophobic interactions with Trp-232, Phe-303,Phe-307, Met-986, and Gln-990 while hydrogen bonding with Gln-990.

Table S4. Structure and activity data of derivatives of propafenone with the substituent groups at R1 and R2 along with their IC50 values for ABCB1 and ABCG2.

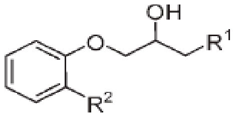

(R1)

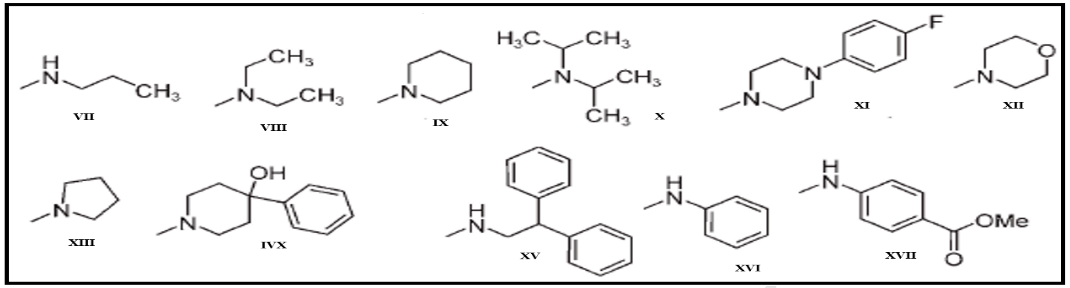

Compounds	R1	R2	IC_{50}(ABCB1) [µM]	IC_{50}(ABCG2) [µM]
36I	VII	$COCH_2CH_2Ph$	1.51	37.9
37I	VIII	$COCH_2CH_2Ph$	1.70	33.5
38I	IX	$COCH_2CH_2Ph$	0.80	30.7
39I	IX	CH_2Ph	1.56	31.9
40I	X	$COCH_2CH_2Ph$	0.65	36.4
41I	X	$CH(OCH_3)CH_2CH_2Ph$	0.65	15.2
42I	X	$CH(OH)CH_2CH_2Ph$	3.01	37.3
43I	XI	$COCH_2CH_2Ph$	0.27	4.62

44I	XI	$CH(OCH_3)CH_2CH_2Ph$	0.09	13.9
45I	XI	COPh	0.79	14.1
46I	XII	$COCH_2CH_3$	113	440
47I	XII	$COCH_2CH_2Ph$	5.83	34.0
48I	XIII	$COCH_2CH_2Ph$	3.60	47.1
49I	IVX	$COCH_3$	1.79	600
50I	IVX	$COCH_2CH_3$	0.68	58.8
51I	IVX	COPh	0.16	98.3
52I	XV	$COCH_2CH_2Ph$	0.39	3.30
53I	XVI	$COCH_2CH_2Ph$	5.50	4.21
54I	XVII	$COCH_2CH_2Ph$	9.61	1.50

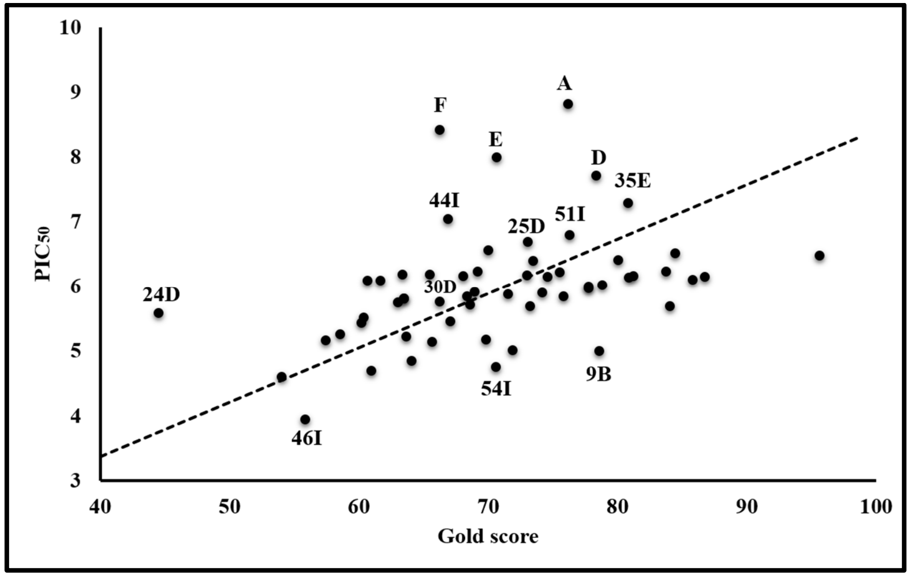

Figure S5. Correlation of inhibitory potency of the dataset (expressed as pIC_{50} values) and Gold score, calculated by docking analysis using Gold software.

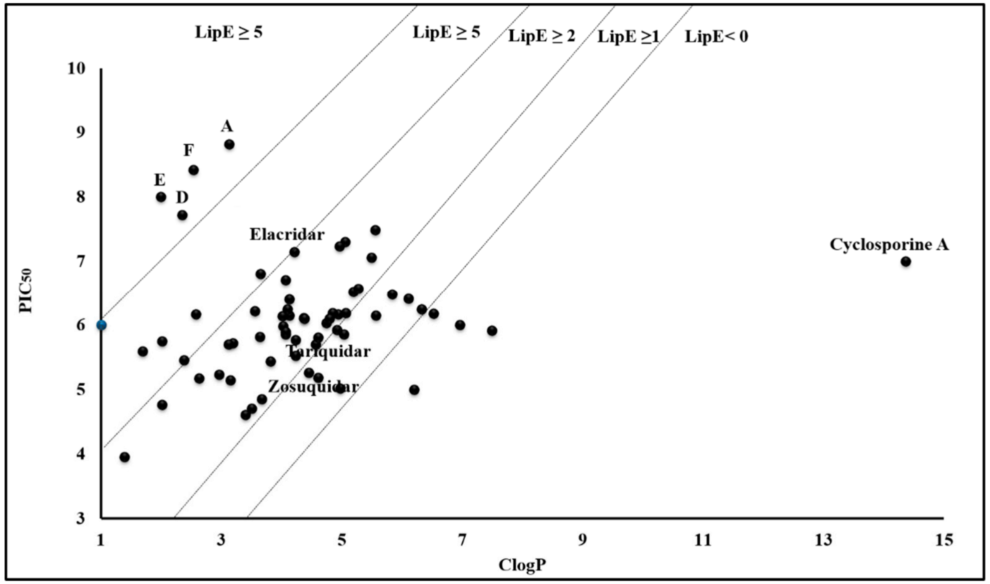

Figure S6. Plot of clogP vs biological activity of the ABCB1-inhibitors dataset and lead compounds along with ; LipE greater than or equal to 5 is considered the threshold for compounds of clinical interest.

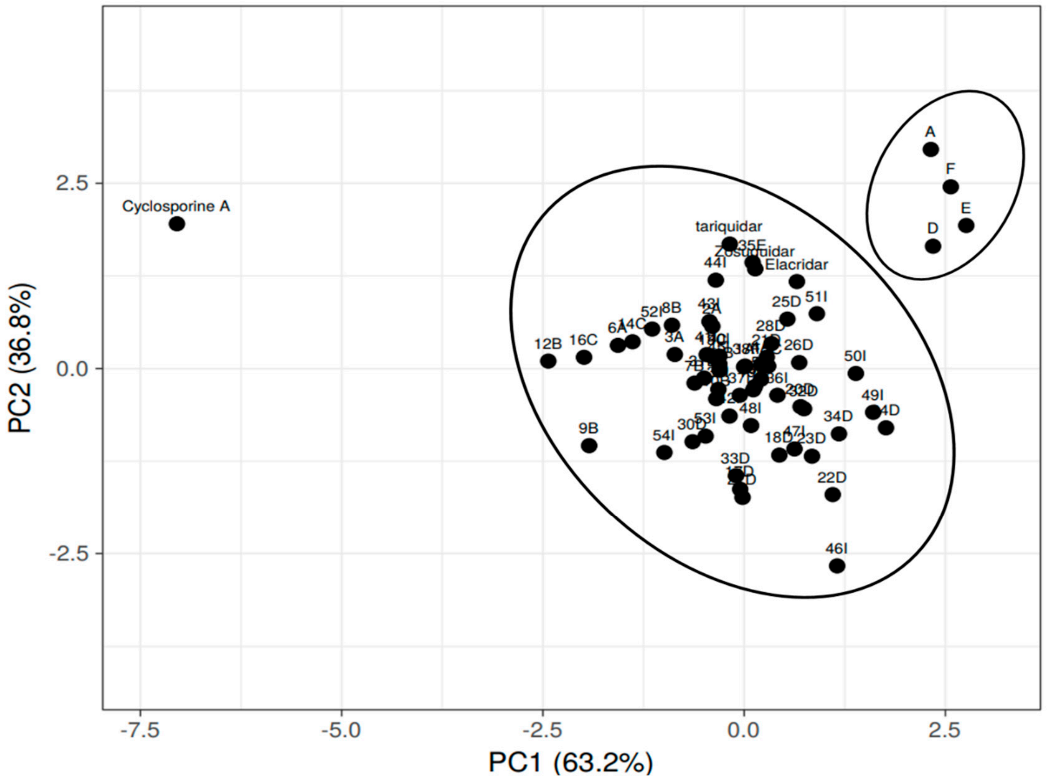

Figure S7. Dataset visualization on the first two principal components (PCs) to find the collinearity between the pIC_{50}, clogP and LipE values of the datasets. The eclipses are drawn for the discrimination of two groups.

Table S5. Comparison of ligand-protein interactions of lead compounds 'A', 'D', 'E', and 'F' complexed with ABCB1 at 0 ns and after 100 ns of MD simulations.

	**Before MD simulations**			**After MD simulations**		
**Protein-ligand complexes**	Hydrophobic	Hydrogen bonding	π stacking	Hydrophobic	Hydrogen bonding	π stacking
**A-ABCB1**	Leu-65 (3.8 Å)		-	Leu-65 (3.8 Å)		-
	Ile-340 (3.6 Å)	Glu-875 (2.1 Å)	-	Ile-340 (3.6 Å)	Glu-875 (2.1 Å)	-
	Phe-343 (3.9 Å)	-	-	Phe-343 (3.9 Å)	-	-
	Ala-871 (3.9 Å)	-	-	Ala-871 (3.9 Å)	-	-
**D-ABCB1**	Phe-336 (3.7 Å)	Gln-725 (2.3 Å)	Phe-303 (5.0 Å)	Phe-336 (3.7 Å)	Gln-725 (2.3 Å)	Phe-303 (5.0 Å)
	Leu-339 (3.7 Å)	Gln-990 (2.7 Å)	-	Leu-339 (3.7 Å)	Gln-990 (2.7 Å)	-
	Ile-340 (3.2 Å)	-	-	Ile-340 (3.2 Å)	-	-
	Phe-983 (3.5 Å)	-	-	Phe-983 (3.5 Å)	-	-
	Phe-728 (2.8 Å)			Phe-728 (2.8 Å)		
**E-ABCB1**	Trp-232 (3.5 Å)	Glu-875 (2.1 Å)	Phe-303 (3.6 Å)	Trp-232 (3.5 Å)	Glu-875 (2.1 Å)	Phe-303 (3.6 Å)
	Phe-303 (3.8 Å)	-	-	Phe-303 (3.8 Å)	-	-

	Tyr-307 (3.8) Å)<br>Phe-343 (3.9 Å)<br>Leu-724 (3.9 Å)	-<br>-<br>-	-<br>-<br>-	Tyr-307 (3.8) Å)<br>Phe-343 (3.9 Å)<br>Leu-724 (3.9 Å)	-<br>-<br>-	-<br>-<br>-
**F-ABCB1**	Trp-232 (4.0 Å)<br>Ile-299 (3.9 Å)<br>Phe-303 (3.8 Å)<br>Ile-306 (3.8 Å)<br>Ile-340 (3.4 Å)<br>Phe-343 (3.8 Å)<br>Met-986 (3.9 Å)<br>Gln-347 (1.9 Å)	Glu-875 (2.5 Å)<br>-<br>-<br>-<br>-<br>-<br>-<br>-	-<br>-<br>-<br>-<br>-<br>-<br>-<br>-	Trp-232 (4.0 Å)<br>Ile-299 (3.9 Å)<br>Phe-303 (3.8 Å)<br>Ile-306 (3.8 Å)<br>Ile-340 (3.4 Å)<br>Phe-343 (3.8 Å)<br>Met-986 (3.9 Å)<br>Gln-347 (1.9 Å)	Glu-875 (2.5 Å)<br>-<br>-<br>-<br>-<br>-<br>-<br>-	-<br>-<br>-<br>-<br>-<br>-<br>-<br>-

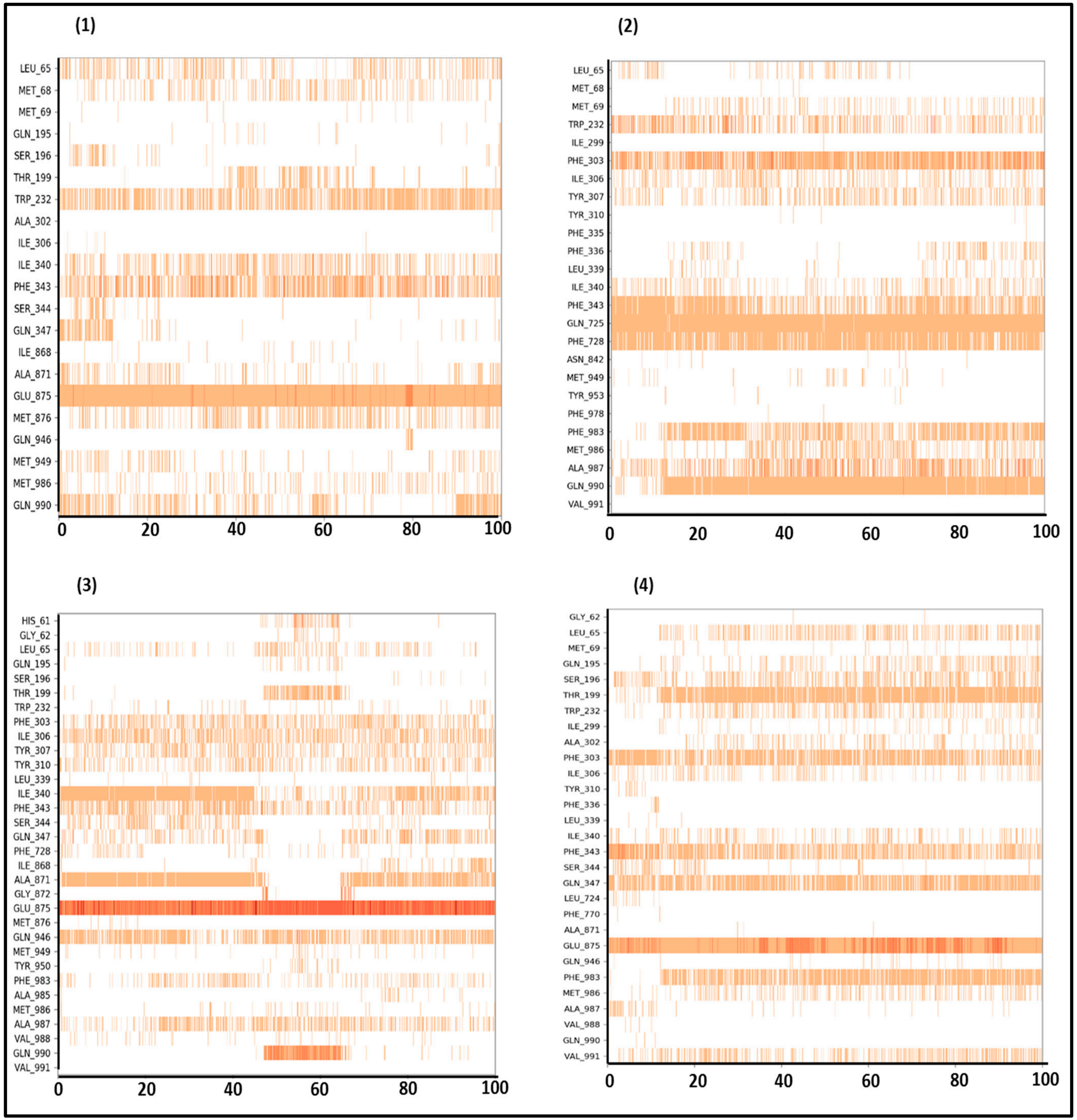
(1)
LEU_65
MET_68
MET_69
GLN_195
SER_196
THR_199
TRP_232
ALA_302
ILE_306
ILE_340
PHE_343
SER_344
GLN_347
ILE_868
ALA_871
GLU_875
MET_876
GLN_946
MET_949
MET_986
GLN_990
0 20 40 60 80 100
(2)
LEU_65
MET_68
MET_69
TRP_232
ILE_299
PHE_303
ILE_306
TYR_307
TYR_310
PHE_335
PHE_336
LEU_339
ILE_340
PHE_343
GLN_725
PHE_728
ASN_842
MET_949
TYR_953
PHE_978
PHE_983
MET_986
ALA_987
GLN_990
VAL_991
0 20 40 60 80 100
(3)
HIS_61
GLY_62
LEU_65
GLN_195
SER_196
THR_199
TRP_232
PHE_303
ILE_306
TYR_307
TYR_310
LEU_339
ILE_340
PHE_343
SER_344
GLN_347
PHE_728
ILE_868
ALA_871
GLY_872
GLU_875
MET_876
GLN_946
MET_949
TYR_950
PHE_983
ALA_985
MET_986
ALA_987
VAL_988
GLN_990
VAL_991
0 20 40 60 80 100
(4)
GLY_62
LEU_65
MET_69
GLN_195
SER_196
THR_199
TRP_232
ILE_299
ALA_302
PHE_303
ILE_306
TYR_310
PHE_336
LEU_339
ILE_340
PHE_343
SER_344
GLN_347
LEU_724
PHE_770
ALA_871
GLU_875
GLN_946
PHE_983
MET_986
ALA_987
VAL_988
GLN_990
VAL_991
0 20 40 60 80 100

Figure S8. Protein-ligand contacts during simulation period of 100 ns where Y-axis represents interacting amino acids of ABCB1 and X-axis represents time of simulation in ns. (1) Protein-ligand contacts in each trajectory frame during the MD-simulation of 100 ns for lead compound 'A' complex with ABCB1. **(2)** Protein-ligand contacts in each trajectory frame during the MD-simulation of 100 ns for lead compound 'D' complex with ABCB1. **(3)** Protein-ligand contacts in each trajectory frame during the MD-simulation of 100 ns for lead compound 'E' complex with ABCB1. **(4)** Protein-ligand contacts in each trajectory frame during the MD-simulation of 100 ns for lead compound 'F' complex with ABCB1.

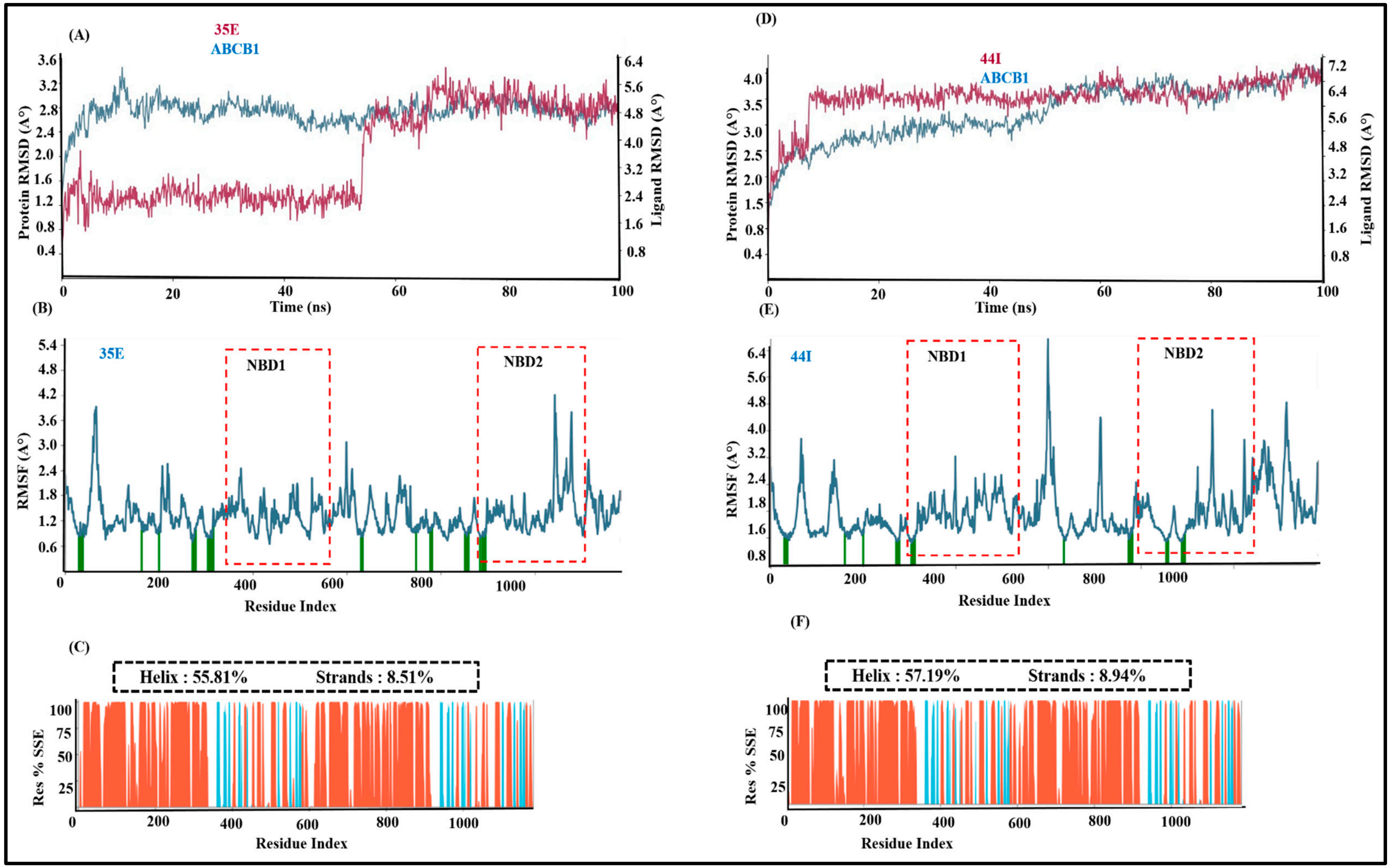

Figure S9. **(A)** RMSD of '35E-ABCB1'complex, **(B)** RMSF of '35E-ABCB1'complex. **(C)** SSE plot of '35E-ABCB1'complex. **(D)** RMSD of '44I-ABCB1'complex, **(E)** RMSF of '44I-ABCB1'complex. **(F)** SSE plot of '44I-ABCB1'complex.

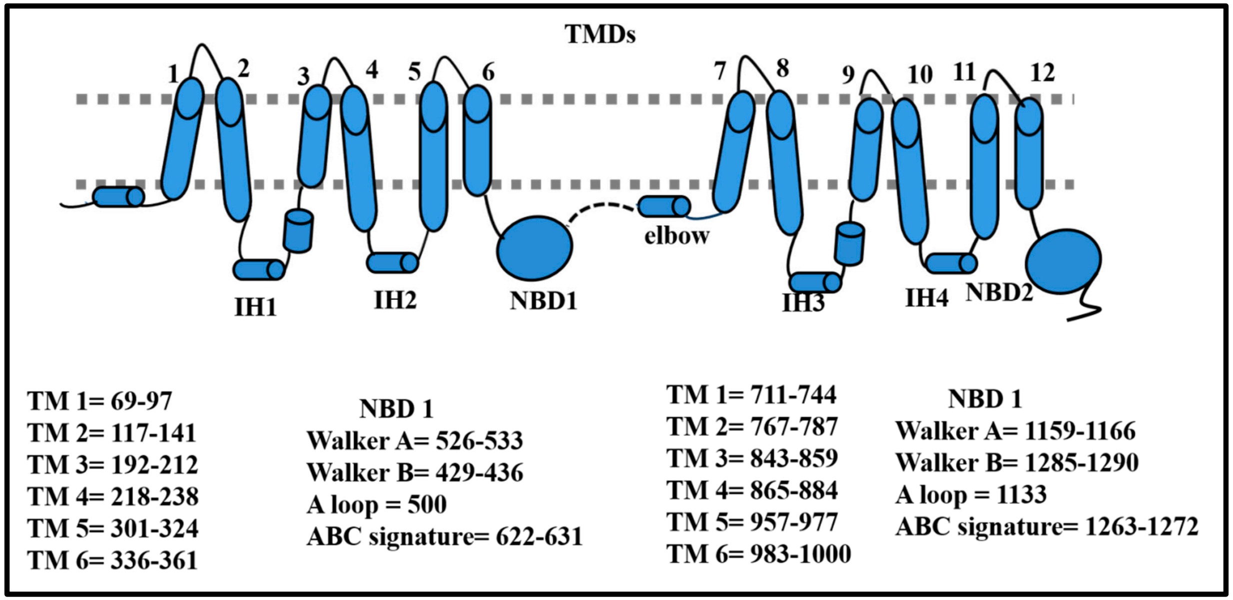

Figure S10. Structural topology of ABCB1 ,consisting of two trans membrane domains (TMD s)and two nucleotide binding domains (NBDs) along with the amino acids numbers.

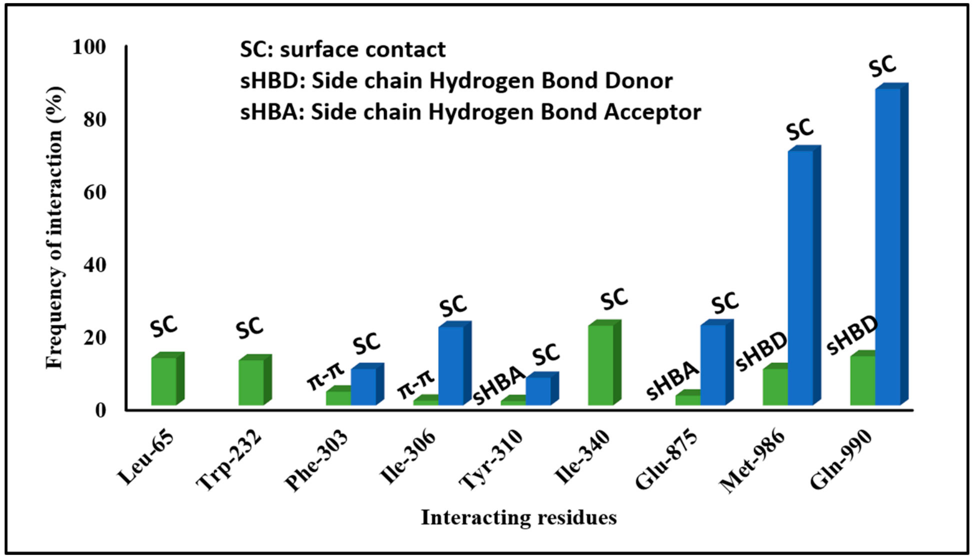

Figure S11. A population histogram based upon the interaction frequency of the ligands and the receptor protein. Most of the residues formed surface contact (hydrophobic and π-π interaction) whereas, some were involved in side-chain hydrogen bond donor and acceptor interactions. Blue and green color bars are just for the presentation of different nature of interaction of the same amino acids.

References

1. Teodori, E.; Dei, S.; Bartolucci, G.; Perrone, M.G.; Manetti, D.; Romanelli, M.N.; Contino, M.; Colabufo, N.A. Structure–Activity Relationship Studies on 6, 7-Dimethoxy-2-phenethyl-1, 2, 3, 4-tetrahydroisoquinoline Derivatives as Multidrug Resistance Reversers. *ChemMedChem* **2017**, *12*, 1369-1379.
2. Jabeen, I.; Pleban, K.; Rinner, U.; Chiba, P.; Ecker, G.F. Structure–activity relationships, ligand efficiency, and lipophilic efficiency profiles of benzophenone-type inhibitors of the multidrug transporter P-glycoprotein. *Journal of medicinal chemistry* **2012**, *55*, 3261-3273.
3. Pajeva, I.K.; Globisch, C.; Wiese, M. Combined pharmacophore modeling, docking, and 3D QSAR studies of ABCB1 and ABCC1 transporter inhibitors. *ChemMedChem: Chemistry Enabling Drug Discovery* **2009**, *4*, 1883-1896.
4. Pellicani, R.Z.; Stefanachi, A.; Niso, M.; Carotti, A.; Leonetti, F.; Nicolotti, O.; Perrone, R.; Berardi, F.; Cellamare, S.; Colabufo, N.A. Potent galloyl-based selective modulators targeting multidrug resistance associated protein 1 and P-glycoprotein. *Journal of medicinal chemistry* **2012**, *55*, 424-436.
5. Cramer, J.; Kopp, S.; Bates, S.E.; Chiba, P.; Ecker, G.F. Multispecificity of drug transporters: probing inhibitor selectivity for the human drug efflux transporters ABCB1 and ABCG2. *ChemMedChem: Chemistry Enabling Drug Discovery* **2007**, *2*, 1783-1788.
6. Cheema, Y.; Kiani, Y.S.; Linton, K.J.; Jabeen, I. Identification and Empiric Evaluation of New Inhibitors of the Multidrug Transporter P-Glycoprotein (ABCB1). *International Journal of Molecular Sciences* **2023**, *24*, 5298.
7. Silbermann, K.; Li, J.; Namasivayam, V.; Baltes, F.; Bendas, G.; Stefan, S.M.; Wiese, M. Superior pyrimidine derivatives as selective ABCG2 inhibitors and broad-spectrum ABCB1, ABCC1, and ABCG2 antagonists. *Journal of Medicinal Chemistry* **2020**, *63*, 10412-10432.
8. Xia, C.Q.; Liu, N.; Miwa, G.T.; Gan, L.-S. Interactions of cyclosporin a with breast cancer resistance protein. *Drug metabolism and disposition* **2007**, *35*, 576-582.
9. Kühnle, M.; Egger, M.; Müller, C.; Mahringer, A.; Bernhardt, G.n.; Fricker, G.; König, B.; Buschauer, A. Potent and selective inhibitors of breast cancer resistance protein (ABCG2) derived from the p-glycoprotein (ABCB1) modulator tariquidar. *Journal of medicinal chemistry* **2009**, *52*, 1190-1197.
